# An Invariant Measure for Differential Entropy: From Kullback–Leibler Divergence to Scale-Invariant Information Theory

**DOI:** 10.3390/e28030301

**Published:** 2026-03-07

**Authors:** Félix Truong, Alexandre Giuliani

**Affiliations:** 1 Synchrotron SOLEIL, L’Orme des Merisiers, 91190 Saint-Aubin, France; alexandre.giuliani@synchrotron-soleil.fr; 2 UAR1008, Transform Department, INRAE, 44316 Nantes, France

**Keywords:** differential entropy, invariant measure, Kullback–Leibler divergence, limiting density of discrete points, k-nearest neighbor, mutual information, scale invariance

## Abstract

Shannon’s differential entropy for continuous variables suffers from a fundamental limitation: it is not invariant under scale transformations. This makes entropy values dependent on the choice of measurement units rather than reflecting intrinsic properties of distributions. While Jaynes proposed the limiting density of discrete points (LDDP) as a theoretical solution, a concrete method for computing the required invariant measure has been lacking. This paper establishes a rigorous connection between Kullback–Leibler divergence and the invariant measure, providing theoretical proofs of invariance under affine transformations and a practical computational method. We prove that entropy normalized by the median of k-nearest neighbor distances is invariant under affine transformations (Theorems 1 and 2). The non-negativity of the resulting entropy has been validated empirically across all tested distribution families, though a complete theoretical proof remains an open question. This approach extends naturally to multivariate settings, enabling scale-invariant mutual information estimation. We provide open-source implementations in Julia (EntropyInvariant.jl) and Python (entropy_invariant) and demonstrate their advantages over traditional approaches, particularly for variables with disparate scales.

## 1. Introduction

Information theory provides powerful tools for quantifying relationships between variables across scientific disciplines. Among these, mutual information (MI) stands out for its ability to capture both linear and nonlinear dependencies while remaining robust to small sample sizes [1,2,3]. Unlike correlation-based measures, MI is sensitive to the complete dependence structure between variables, making it particularly valuable for complex data analysis [4].

The foundation of MI lies in Shannon’s entropy framework [5], originally defined for discrete variables and later extended to continuous variables as differential entropy. For a continuous random variable *X* with probability density function $\mu_{X}$, the differential entropy is:

(1) $$h\left(X\right)=-\int_{X}\mu_{X}\left(x\right)\cdot \log\left(\mu_{X}\left(x\right)\right)\cdot dx$$

This definition applies to both bounded and unbounded domains, provided the integral converges. Our invariant measure methodology requires that the median of *k*-nearest neighbor distances stabilizes with increasing sample size, a condition empirically satisfied even for heavy-tailed distributions such as Cauchy and Lévy (Table 2).

However, differential entropy suffers from a critical limitation: it is not invariant under scale transformations. Under a linear transformation Y=aX, the entropy transforms as:

(2) h(aX)=h(X)+log(|a|)

This scale-dependence means that entropy values depend on measurement units rather than reflecting intrinsic properties of the distribution. For instance, measuring temperature in Celsius versus Fahrenheit yields different entropy values, even though the underlying physical system is identical. This limitation extends to mutual information, potentially leading to misleading results when comparing variables with different scales.

MI is zero when *X* and *Y* are independent and increases with the dependence between variables. This measure is always non-negative and has no upper bound, expressed as MI(X,Y)≥0. MI can be expressed in terms of differential entropy as:

(3) MI(X;Y)=h(X)+h(Y)−h(X,Y)

where h(X,Y) denotes the joint differential entropy. Equivalently, $MI\left(X;Y\right)=D_{KL}\left(P_{X,Y}∥P_{X}⊗P_{Y}\right)$, measuring the KL divergence from the joint distribution to the product of marginals. While MI shares some metric-like properties (non-negativity, symmetry), it does not satisfy the triangle inequality and is therefore not a true metric in the mathematical sense; we use the term “measure of dependence” throughout.

A significant challenge in many practical applications lies in estimating entropy from finite samples when the underlying probability density function (pdf) is unknown. Techniques for entropy estimation can be broadly classified into two main categories: parametric and nonparametric methods [6]. Parametric methods assume that the form of the pdf is known, reducing the problem to estimating the parameters from the data [1]. Nonparametric methods do not make assumptions about the form of the pdf, making them more versatile and widely applicable. Among these, popular approaches include histogram-based methods [7], kernel density estimators (KDEs) [8], and entropy-based statistical tests [9]. Another nonparametric approach is based on the k-nearest neighbors (kNN) method [3,6], which is computationally efficient and has been shown to be robust even with small sample sizes [10,11].

Despite their popularity, traditional nonparametric methods have significant limitations. Histogram-based methods are highly sensitive to the choice of bin width: too few bins lead to oversmoothing and information loss, while too many bins result in high variance and systematic bias that increases with the number of bins. The optimal bin width is data-dependent and difficult to determine a priori. KDE methods face similar bandwidth selection challenges. While the kNN method addresses some of these issues by using a single, interpretable parameter *k* and demonstrating good convergence properties, it remains sensitive to scale transformations. For comprehensive coverage of information-theoretic foundations, we refer readers to [12].

The root of the scale-dependence problem lies in the transition from discrete to continuous entropy. To understand this, consider the Kullback–Leibler (KL) divergence [13], which measures the dissimilarity between distributions *P* and *Q*:

(4) $$D_{KL}\left(P||Q\right)=\int_{X}\mu_{P}\left(x\right)\cdot \log\left(\frac{\mu_{P}\left(x\right)}{\mu_{Q}\left(x\right)}\right)\cdot dx$$

The KL divergence relates to differential entropy through:

(5) $$D_{KL}\left(P||Q\right)=-h\left(P\right)-E_{P}\left[\log\left(\mu_{Q}\left(X\right)\right)\right]$$

When *Q* is a uniform distribution over an interval of length *r*, we obtain:

(6) $$h\left(P\right)=\log\left(r\right)-D_{KL}\left(P|\right|U_{r})$$

This reveals that differential entropy implicitly compares the distribution to a uniform reference whose scale affects the entropy value. The scale-dependence arises because the reference scale *r* changes under transformations.

Jaynes [14] recognized this fundamental issue and proposed addressing it through an “invariant measure” m(x) representing complete ignorance about the probability distribution. This leads to the limiting density of discrete points (LDDP):

(7) $$H_{c}\left(X\right)=-\int_{X}\mu_{X}\left(x\right)\cdot \log\left(\frac{\mu_{X}\left(x\right)}{m(x)}\right)\cdot dx$$

Comparing Equations (4) and (7), we observe that the LDDP can be interpreted as a KL divergence where the reference distribution is replaced by the invariant measure. However, Jaynes did not provide a concrete method for computing m(x), leaving the central question unanswered: what properties must m(x) satisfy, and how can it be estimated from data?

Recent work by Nagel and coworkers [15] proposed normalizing MI using an invariant measure, but their approach introduces inconsistencies: the marginal entropies vary depending on which variables are paired together, violating the fundamental principle that a variable’s entropy should be intrinsic to that variable alone.

Our contributions: This paper establishes a rigorous connection between the KL divergence framework and Jaynes’s invariant measure concept. We demonstrate that:

- The invariant measure m(x) can be rigorously defined through specific mathematical properties and computed from data using k-nearest neighbor distances;
- This measure naturally emerges from requiring transformation invariance analogous to the KL divergence framework;
- The resulting invariant entropy corresponds to the LDDP and is truly scale-invariant;
- The approach generalizes naturally to multivariate settings, enabling consistent scale-invariant MI estimation;
- The median of nearest neighbor distances provides a robust estimator that avoids negative entropy values and identifies distribution families.

The present manuscript is organized as follows. Section 2 develops the theoretical framework connecting KL divergence to the invariant measure, proves invariance properties, and presents the computational method. Section 3 validates the approach through simulations and demonstrates its advantages over traditional methods. Section 4 discusses connections to information geometry, maximum entropy principles, and broader implications. Section 5 concludes with future directions.

## 2. Materials and Methods

### 2.1. From Kullback–Leibler Divergence to Invariant Measure

To understand the connection between KL divergence and invariant entropy, we examine what happens when comparing a distribution to a uniform reference. We use a bounded interval here for pedagogical clarity; the resulting invariant measure framework applies broadly to distributions with bounded or unbounded support, as demonstrated in Section 3. Consider a uniform distribution $U_{\left[a,b\right]}$ with density $\mu_{U}\left(x\right)=1/\left(b-a\right)$ for x∈[a,b].

For a distribution *P* with support contained in [a,b], the KL divergence is:

(8) $$D_{KL}\left(P|\right|U_{\left[a,b\right]})=-h\left(P\right)+\log\left(b-a\right)$$

Under an affine transformation Y=aX+b, the uniform reference transforms to $U_{\left[aa+b,ab+b\right]}$ with density 1/(|a|(b−a)). The KL divergence becomes:

(9) $$D_{KL}(P_{Y}\left|\right|U_{Y})=-h\left(X\right)+\log\left(b-a\right)=D_{KL}(P_{X}\left|\right|U_{X})$$

This demonstrates that the KL divergence to a uniform reference is invariant under affine transformations, but differential entropy is not because the log-volume term changes. The key insight is that we need a reference measure that adapts to the data scale in a way that removes this scale-dependence.

### 2.2. Definition of the Invariant Measure

We propose that the invariant measure m(x) should satisfy properties that ensure scale invariance while remaining practically computable from data.

**Proposition** **1.** *Let $m:R\to R^{+}$ be an invariant measure function. We require:*

*(i)*     *Positivity: $m\left(X\right)=r_{X}>0$ for any random variable X;*
*(ii)*    *Scale equivariance: m(aX)=|a|·m(X) for any a≠0;*
*(iii)*   *Translation invariance: m(X+b)=m(X) for any b∈R;*
*(iv)*   *Consistency with KL divergence: The measure should lead to an entropy-like quantity that behaves as a KL divergence from the data distribution to a reference distribution.*

These properties ensure that m(X) captures the intrinsic scale of the distribution independent of affine transformations. We now define the invariant differential entropy as:

(10) $$h_{c}\left(X\right):=h\left(\frac{X}{m(X)}\right)$$

**Theorem** **1** (Invariance of $h_{c}$)**.** *Let m(X) satisfy Properties (i)–(iii) of Proposition 1. Then, for any transformation Y=aX+b with a≠0:*

(11) $$h_{c}\left(Y\right)=h_{c}\left(X\right)$$

**Proof.**  Let Y=aX+b. Based on Properties (iii) and (ii), we have:

(12) m(aX+b)=m(aX)=|a|·m(X)

Therefore:

(13) $$h_{c}\left(Y\right)=h\left(\frac{Y}{m(Y)}\right)=h\left(\frac{aX+b}{\left|a|\cdot m(X\right)}\right)$$

Let Z=X/m(X) be the normalized variable. Under the transformation Y=aX+b, we have:

(14) $$\frac{Y}{m(Y)}=\frac{aX+b}{\left|a|\cdot m(X\right)}=\mathrm{sgn}\left(a\right)\cdot \frac{X}{m(X)}+\frac{b}{\left|a|\cdot m(X\right)}=\mathrm{sgn}\left(a\right)\cdot Z+c$$

where c=b/(|a|·m(X)) is a constant and sgn(a)=±1.

Now, we apply the change-of-variables formula for differential entropy. For a random variable *Z* with density $\mu_{Z}\left(z\right)$ and a transformation W=g(Z) where *g* is differentiable and invertible, the density of *W* is:

(15) $$\mu_{W}\left(w\right)=\mu_{Z}\left(g^{-1}\left(w\right)\right)\cdot \left|\frac{dg^{-1}}{dw}\right|$$

For a linear transformation W=aZ+c, we have $g^{-1}\left(w\right)=\left(w-c\right)/a$ and $|dg^{-1}/dw|=1/|a|$, giving:

(16) $$h\left(aZ+c\right)=-\int \mu_{W}\left(w\right)\log\left(\mu_{W}\left(w\right)\right)dw=-\int \frac{1}{\left|a\right|}\mu_{Z}\left(\frac{w-c}{a}\right)\log\left(\frac{1}{\left|a\right|}\mu_{Z}\left(\frac{w-c}{a}\right)\right)dw$$

Substituting u=(w−c)/a, dw=|a|du:

(17) $$h\left(aZ+c\right)=-\int \mu_{Z}\left(u\right)\log\left(\frac{1}{\left|a\right|}\mu_{Z}\left(u\right)\right)\left|a\right|\frac{du}{\left|a\right|}=-\int \mu_{Z}\left(u\right)[\log\left(\mu_{Z}\left(u\right)\right)-\log(|a|)]du$$

(18) $$=-\int \mu_{Z}\left(u\right)\log\left(\mu_{Z}\left(u\right)\right)du+\log\left(|a|\right)\int \mu_{Z}\left(u\right)du=h\left(Z\right)+\log\left(|a|\right)$$

However, for our normalized variable, a=sgn(a) has |a|=1, so:

(19) $$h\left(\mathrm{sgn}(a)\cdot Z+c\right)=h\left(Z\right)+\log\left(1\right)=h\left(Z\right)=h\left(\frac{X}{m(X)}\right)=h_{c}\left(X\right)$$

Therefore, $h_{c}\left(Y\right)=h_{c}\left(X\right)$, establishing invariance.    □

### 2.3. Connection to Jaynes’s LDDP

We now show that $h_{c}\left(X\right)$ is equivalent to Jaynes’s LDDP. Using the change of variables u=x/m(X), we have x=m(X)·u and dx=m(X)·du. The density transforms as:

(20) $$\mu_{X/m(X)}\left(u\right)=m\left(X\right)\cdot \mu_{X}\left(m\left(X\right)\cdot u\right)$$

Therefore:

(21) $$\begin{array}{cc} h_{c}\left(X\right) & =h\left(\frac{X}{m(X)}\right)=-\int \mu_{X/m(X)}\left(u\right)\log\left(\mu_{X/m(X)}\left(u\right)\right)du\\ \; & =-\int m\left(X\right)\mu_{X}\left(m\left(X\right)u\right)\log\left(m\left(X\right)\mu_{X}\left(m\left(X\right)u\right)\right)du\\ \; & =-\int \mu_{X}\left(x\right)\log\left(m\left(X\right)\mu_{X}\left(x\right)\right)\frac{dx}{m(X)}\\ \; & =-\int \mu_{X}\left(x\right)\left[\log\left(\mu_{X}\left(x\right)\right)+\log\left(m\left(X\right)\right)\right]dx\\ \; & =h(X)-\log(m(X)) \end{array}$$

This can be expressed as:

(22) $$h_{c}\left(X\right)=-\int \mu_{X}\left(x\right)\log\left(\frac{\mu_{X}\left(x\right)}{1/m(X)}\right)dx$$

which is precisely Jaynes’s expression (7) with constant invariant measure m(x)=1/m(X). Hence, as mentioned in the Introduction, the invariant entropy corresponds to comparing the distribution to a uniform reference over an interval of length m(X), which adapts to the data scale.

### 2.4. Estimation of the Invariant Measure

To make this framework practical, we need a method to estimate m(X) from data. We propose using the k-nearest neighbor (kNN) approach, which aligns naturally with kNN-based entropy estimation methods.

Given a sample $\left\{x_{1},x_{2},\ldots ,x_{n}\right\}$ from *X*, we compute the distance from each point to its *k*-th nearest neighbor:

(23) $$d_{i}^{\left(k\right)}=\min_{j\neq i}^{\left(k\right)}\left|x_{i}-x_{j}\right|,\;i=1,\ldots ,n$$

where $\min^{\left(k\right)}$ denotes the *k*-th smallest value. For simplicity, we focus on k=1 (nearest neighbor). The vector of nearest neighbor distances $d=(d_{1},\ldots ,d_{n})$ captures the local density structure. We propose:

(24) m(X)=median(d)

Justification for using the median:

(1) Robustness: The median is robust to outliers, reflecting “complete ignorance” about points far from the data bulk. Consider X={2,3,5,7,11,17,19,23,29} with nearest neighbor vector d={1,1,2,2,4,2,2,4,6} (sorted: {1,1,2,2,2,2,4,4,6}), giving m(X)=2 and mean(d)=2.67. Adding an outlier $x_{10}=100$ yields $d_{10}=\left|100-29\right|=71$ and $d^{\prime }=\left\{1,1,2,2,4,2,2,4,6,71\right\}$, still giving $m(X^{\prime })=2$. The mean would change from 2.67 to 9.50, demonstrating inferior robustness.

(2) Scale equivariance: For scaled data aX, nearest neighbor distances scale as $\left|a\right|d_{i}$, so median(|a|d)=|a| median(d), satisfying Property (ii) of Proposition 1.

(3) Translation invariance: Translating data by *b* does not change distances, so median(d) remains unchanged, satisfying Property (iii).

(4) Avoids negative entropy: Using the mean can lead to negative entropy. For example, the exponential and normal distributions yield negative values with the mean-based measure (Table 1):

Empirically, the median avoids negative entropy values while preserving the desired invariance properties. This robustness likely stems from the median’s optimality as an $L^{1}$ center, which is less sensitive to extreme values in the distance distribution than the mean ($L^{2}$ center). A rigorous proof establishing conditions under which the median measure guarantees non-negative entropy for all distribution classes remains an important open theoretical question.

Theoretical status: Our main results (Theorems 1 and 2) rigorously establish that the median-based invariant measure satisfies scale equivariance and translation invariance. The non-negativity of $h_{c}\left(X\right)$ has been verified empirically for all distribution families in Table 2, but a general proof guaranteeing $h_{c}\left(X\right)\geq 0$ for all distributions remains an important open question.

### 2.5. Multivariate and Multidimensional Generalization

We distinguish between the multivariate setting (multiple random variables $X_{1},\ldots ,X_{n}$, each potentially vector-valued) and the multidimensional setting (a single variable $X\in R^{d}$). For a multidimensional variable, m(X) is computed from kNN distances in $R^{d}$. For multiple variables, each $m(X_{i})$ is computed from the marginal distribution independently, and the joint measure uses the product form $m\left(X_{1},\ldots ,X_{n}\right)=\prod_{i}m\left(X_{i}\right)$.

The extension to multiple variables follows naturally from the KL divergence perspective. For two random variables (X,Y), the joint LDDP is:

(25) $$H_{c}\left(X,Y\right)=-\int \int \mu_{X,Y}\left(x,y\right)\log\left(\frac{\mu_{X,Y}\left(x,y\right)}{m(x,y)}\right)dxdy$$

**Proposition** **2** (Separable invariant measure)**.** *For independent scale transformations, the invariant measure for the joint distribution should satisfy:*

(26) $$m\left(x,y\right)=m_{X}\left(x\right)\cdot m_{Y}\left(y\right)$$

*where $m_{X}$ and $m_{Y}$ are the marginal invariant measures.*

**Theorem** **2** (Joint invariant entropy)**.** *Let (X,Y) be jointly distributed random variables with joint density $\mu_{X,Y}$, marginal densities $\mu_{X}$ and $\mu_{Y}$, and marginal invariant measures m(X) and m(Y) computed from the respective marginal samples. Define the normalized variables $X^{\prime }=X/m\left(X\right)$ and $Y^{\prime }=Y/m\left(Y\right)$. The invariant joint entropy is:*

(27) $$h_{c}\left(X,Y\right)=h\left(\frac{X}{m(X)},\frac{Y}{m(Y)}\right)$$

*This is invariant under independent affine transformations $\left(X,Y\right)\to \left(a_{1}X+b_{1},a_{2}Y+b_{2}\right)$, for any $a_{1},a_{2}\neq 0$ and $b_{1},b_{2}\in R$.*

Geometric interpretation via kNN distances:

The kNN entropy estimator in 2D uses Euclidean distances. For points $A=(x_{i},y_{i})$ and $B=(x_{j},y_{j})$:

(28) $$d\left(A,B\right)=\sqrt{{\left(x_{i}-x_{j}\right)}^{2}+{\left(y_{i}-y_{j}\right)}^{2}}$$

Under uniform transformation (x,y)→(kx,ky), distances scale as d→kd. However, for independent transformations $\left(x,y\right)\to \left(k_{1}x,k_{2}y\right)$:

(29) $$d\left(k_{1}A,k_{2}B\right)=\sqrt{k_{1}^{2}{\left(x_{i}-x_{j}\right)}^{2}+k_{2}^{2}{\left(y_{i}-y_{j}\right)}^{2}}$$

which is not simply proportional to d(A,B).

By normalizing each coordinate by its invariant measure:

(30) $$A^{\prime }=\left(\frac{x_{i}}{m(X)},\frac{y_{i}}{m(Y)}\right),\;B^{\prime }=\left(\frac{x_{j}}{m(X)},\frac{y_{j}}{m(Y)}\right)$$

The distance becomes:

(31) $$\begin{array}{cc} d(k_{1}A^{\prime },k_{2}B^{\prime }) & =\sqrt{{\left(k_{1}\frac{x_{i}}{m(k_{1}X)}-k_{1}\frac{x_{j}}{m(k_{1}X)}\right)}^{2}+{\left(k_{2}\frac{y_{i}}{m(k_{2}Y)}-k_{2}\frac{y_{j}}{m(k_{2}Y)}\right)}^{2}}\\ \; & =\sqrt{{\left(\frac{x_{i}-x_{j}}{m(X)}\right)}^{2}+{\left(\frac{y_{i}-y_{j}}{m(Y)}\right)}^{2}}\\ \; & =d(A^{\prime },B^{\prime }) \end{array}$$

This shows that the normalized coordinates create a natural reference frame where distances are invariant under independent scale transformations—precisely what is needed for invariant entropy estimation.

By normalizing each coordinate by its invariant measure, we create a natural reference frame where distances are invariant under independent scale transformations. The invariant mutual information is then:

(32) $$MI_{c}\left(X,Y\right)=h_{c}\left(X\right)+h_{c}\left(Y\right)-h_{c}\left(X,Y\right)$$

## 3. Results

### 3.1. Validation with Standard Distributions

**Figure 1:**
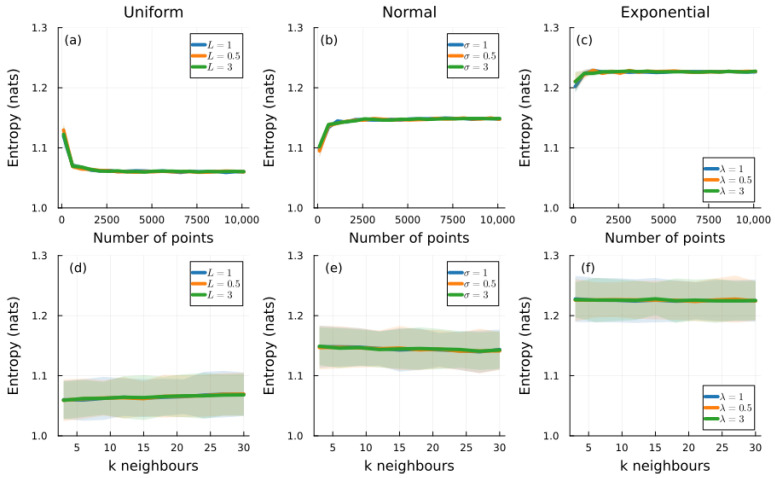
Invariant entropy estimation for: (**a**,**d**) uniform distribution U(0,b) with b=[1,0.5,3]; (**b**,**e**) normal distribution N(0,σ) with σ=[1,0.5,3]; (**c**,**f**) exponential distribution E(λ) with λ=[1,0.5,3]. Top panels show entropy versus sample size; bottom panels show entropy versus number of neighbors *k*. The green shaded area denotes the standard deviation from 100 simulations. The uniform distribution has bounded support [0,b]; the normal and exponential distributions have unbounded support but rapidly decaying tails, ensuring stable estimation of m(X) from finite samples.

To validate our invariant measure approach, we performed extensive simulations comparing it with traditional kNN and histogram methods. The purpose of Figure 1 is to test the core invariance property: distributions differing only in scale parameters should yield identical invariant entropy. Each column corresponds to a different distribution family (uniform, normal, exponential), with three scale parameter values overlaid. The top panels show convergence with sample size, while the bottom panels show stability across the number of neighbors *k*.

The key observation is that distributions with the same shape but different scale parameters yield identical invariant entropy values (overlapping curves in Figure 1). This confirms that the invariant measure successfully removes scale dependence. The convergence is rapid, with the estimation stabilizing at approximately 1500 samples. Moreover, the standard deviation is small and remains consistent across different parameter values, demonstrating the robustness of the approach.

### 3.2. Distribution Identification via Invariant Entropy

The median-based invariant entropy identifies distribution families independent of their location and scale parameters. Table 2 presents invariant entropy values for common continuous distributions.

Table 2 presents invariant entropy values for 14 common distribution families, ordered from lowest to highest. The ordering reflects a spectrum from highly predictable local structure (arcsine, 1.008) to highly unpredictable local structure (Lévy, 1.973). Several observations emerge. First, the invariant entropy uniquely characterizes each distribution family: all normal distributions share the same value (1.150), all exponential distributions share the same value (1.227), etc. The parameter-free nature of the invariant entropy is demonstrated in Figure 1, where curves for different scale parameters (σ=[0.5,1,3]) overlap completely. Second, the arcsine distribution has the lowest invariant entropy (1.008), even lower than the uniform distribution (1.060). This reflects the arcsine distribution’s distinctive property: its probability density concentrates at the boundaries x=a and x=b, where $\mu_{\mathrm{Arcsine}}\left(x\right)=1/\left(\pi \sqrt{\left(x-a)(b-x\right)}\right)$. Given the typical nearest-neighbor spacing captured by m(X), points near the boundaries are highly predictable relative to the invariant measure scale, resulting in lower entropy. Third, heavy-tailed distributions like Cauchy and Levy have higher invariant entropy, reflecting their greater unpredictability at the scale of typical nearest-neighbor distances.

### 3.3. Scale-Invariant Mutual Information

To demonstrate the practical advantage of invariant MI, we simulated three independent variables with vastly different scales: X∼N(0,0.1), Y∼N(0,1), and Z∼N(0,10). These differing standard deviations highlight the advantages of the invariant entropy estimation. Since the variables are independent, the theoretical MI between any pair should be zero.

**Figure 2:**
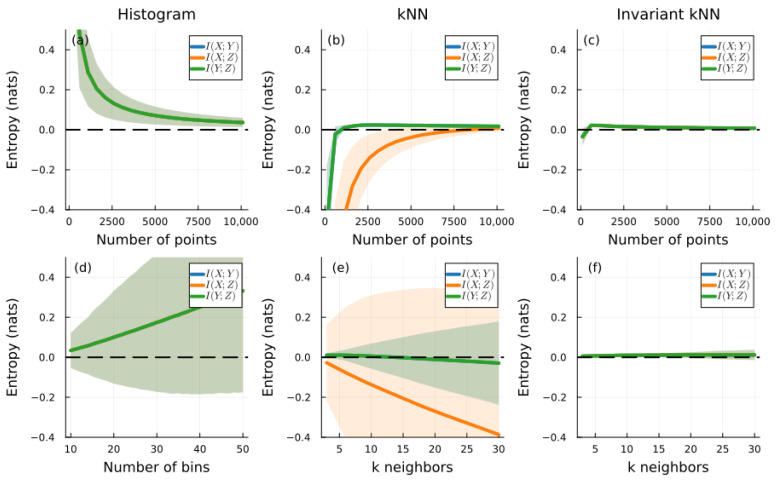
MI estimation between three independent random variables as a function of the number of points (**a**–**c**) and the number of bins/neighbors (**d**–**f**). The histogram method results are shown in (**a**,**d**), the kNN method in (**b**,**e**), and the invariant estimation in (**c**,**f**). The variables follow X∼N(0,0.1), Y∼N(0,1), and Z∼N(0,10). The green shaded area denotes the standard deviation from 100 simulations. All variables are normally distributed with unbounded support; the invariant measure stabilizes due to rapid tail decay despite the 100-fold scale differences. Since the variables are independent, the true MI is zero for all pairs; a successful estimator should yield MI≈0 regardless of the scale difference.

Figure 2 demonstrates striking differences between methods. The histogram method (panels a,d) achieves scale invariance, with all three MI estimates superposed in panel (a). However, convergence toward the theoretical value of zero is extremely slow as sample size increases (panel a), requiring thousands of points to approach the correct value. Furthermore, panel (d) reveals significant systematic bias that varies with the number of bins, making parameter selection critical and problematic. The bias increases substantially with larger bin counts, demonstrating a fundamental limitation of the binning approach.

The kNN method (panels b,e) shows faster convergence than histograms. In panel (b), I(X;Y) and I(Y;Z) are superposed and converge relatively quickly to zero. Panel (e) reveals no bias for k<20—the typical range preferred in the literature where smaller *k* values are standard. However, a severe breakdown emerges for I(X;Z): in panel (e), this estimate diverges toward increasingly negative values, a physical impossibility since mutual information is non-negative by definition. This failure is not a minor numerical artifact but a fundamental problem: the divergence persists across all values of *k* (panel e). The breakdown occurs because the kNN estimator implicitly assumes comparable scales when computing joint nearest-neighbor distances. The 100-fold magnitude difference between *X* (scale ∼ 0.1) and *Z* (scale ∼ 10) causes the *Z* coordinate to completely dominate the Euclidean distance metric, corrupting the joint entropy estimation.

In contrast, the invariant method (panels c,f) demonstrates superior performance across all metrics. In panel (c), all three MI estimates (I(X;Y), I(Y;Z), and I(X;Z)) are perfectly superposed, confirming complete scale invariance regardless of the magnitude differences between variables. The convergence toward zero is faster than both the histogram and kNN methods, achieving accurate estimates with fewer than 2500 samples. Crucially, panel (f) shows no bias regardless of the number of neighbors *k*, eliminating the need for careful parameter tuning. The estimates remain stable and centered near zero across the entire range k∈[3,30]. While finite-sample estimation errors can occasionally produce small negative values near zero due to statistical fluctuations, the invariant method avoids the divergence that afflicts standard kNN estimation.

These simulations establish the superiority of the invariant approach across multiple dimensions. First, it achieves faster convergence than competing methods, requiring fewer samples to reach accurate estimates. Second, it demonstrates complete scale invariance with all MI pairs collapsing to a single curve, independent of scale differences spanning four orders of magnitude (0.1 to 10). Third, it exhibits no parameter-dependent bias, maintaining stability across a wide range of *k* values without requiring careful tuning. Fourth, it avoids the catastrophic divergence to negative values that plague traditional kNN estimation when variables have disparate scales.

### 3.4. Computational Efficiency

The computational complexity of our method is identical to standard kNN entropy estimation: O(NlogN) for sorting and O(N) for nearest neighbor search using KD-trees or ball trees. The additional computation of m(X) via median is O(N), making it negligible compared to the nearest neighbor search. The Julia package EntropyInvariant.jl provides an efficient implementation with performance comparable to standard kNN methods (see the Appendix A for detailed usage examples).

## 4. Discussion

### 4.1. Theoretical Contributions

This work establishes a rigorous connection between Kullback–Leibler divergence and Jaynes’s limiting density of discrete points. Our main theoretical contributions are:

1. Formalization of the invariant measure: We have shown that the invariant measure m(X) can be understood through the lens of KL divergence as a data-adaptive reference scale. When differential entropy is expressed relative to a uniform distribution, the implicit scale factor log(r) introduces scale-dependence. By comparing to a reference distribution with characteristic scale m(X) estimated from the data itself, we remove this implicit scale dependence. This is analogous to using an empirical prior in Bayesian statistics: the reference incorporates information about the typical scale of the phenomenon.
2. Bridge between discrete and continuous entropy: The KL divergence framework naturally connects Shannon’s discrete entropy to differential entropy. Our invariant measure provides the missing piece for making the continuous case behave like the discrete case with respect to invariance properties. Just as Shannon entropy for discrete variables is invariant to relabeling of outcomes, our invariant entropy for continuous variables is invariant to rescaling of measurement units.
3. Rigorous change-of-variables proof: Unlike previous informal arguments, our proof of invariance (Theorem 1) explicitly uses the change-of-variables formula for probability densities. This establishes the result on solid mathematical foundations and clarifies the role of the Jacobian in transformation properties.
4. Multivariate generalization with geometric interpretation: The extension to joint distributions follows naturally from the separability principle in KL divergence (Proposition 2). The geometric interpretation shows that normalizing by marginal invariant measures creates a coordinate system where Euclidean distances are invariant under independent scale transformations, a property essential for multivariate kNN entropy estimation.

### 4.2. Comparison with Existing Approaches

Traditional histogram methods: As demonstrated in the original work and Figure 2, histogram methods suffer from systematic bias that increases with the number of bins. There is no principled way to choose the optimal bin width, and the method shows severe scale sensitivity. Our approach eliminates these issues by using a data-adaptive scale.

Standard kNN estimator: The standard kNN estimator [3] provides consistent entropy estimates and is computationally efficient. However, it is not scale-invariant, as evidenced by the negative MI values in Figure 2d. Our approach modifies this by normalizing data by m(X) before applying the kNN estimator, yielding ${\hat{h}}_{c}\left(X\right)=\hat{h}\left(X/m\left(X\right)\right)$. This simple modification preserves all the advantages of kNN methods while adding scale invariance.

Kernel density estimators: KDE methods [8] face similar bandwidth selection challenges as histogram methods. While sophisticated adaptive bandwidth selection procedures exist, they add computational complexity. Our median-based invariant measure provides a simple, robust alternative that requires no tuning beyond the standard *k* parameter.

Nagel et al.’s approach: Nagel and coworkers [15] proposed normalizing MI by subtracting a scaling factor computed from marginal entropies. However, their normalization has a fundamental flaw: it affects marginal entropies differently depending on which variables are paired together. For example, the normalized entropy of *X* when computed with *Y* differs from its normalized entropy when computed with *Z*. This violates the principle that a variable’s entropy should be an intrinsic property. Our approach provides consistent entropy for each variable regardless of which other variables it is paired with, because each variable has its own invariant measure m(X) computed from its marginal distribution.

### 4.3. Interpretation of Results

Our simulation results demonstrate several key properties:

1. Fast convergence with small samples: The invariant estimator converges faster than traditional methods, particularly when variables have different scales (Figure 2, panels e,f versus c,d). This occurs because normalization by m(X) and m(Y) brings variables to comparable scales before computing joint entropy. The kNN distance calculations then operate in a balanced space where all dimensions contribute equally.
2. Distribution identification: Table 2 shows that distributions maintain consistent invariant entropy values regardless of location and scale parameters. From the KL divergence perspective, this makes sense: distributions in the same family (e.g., all normal distributions) differ only in location parameter μ and scale parameter σ. The invariant measure removes precisely these parameters, leaving only the intrinsic “shape” of the distribution. This property enables distribution classification based solely on shape characteristics.
3. Boundary concentration in arcsine distribution: The arcsine distribution has the lowest invariant entropy (1.008), even lower than uniform (1.060). This initially surprising result reflects a deep property of the arcsine distribution. Its density $\mu \left(x\right)=1/\left(\pi \sqrt{\left(x-a)(b-x\right)}\right)$ diverges at the boundaries, meaning probability mass concentrates there. When we measure entropy relative to the typical nearest-neighbor spacing m(X), points near boundaries are highly predictable, and their neighbors must also be near the boundary. This local predictability, captured by the invariant measure, results in lower entropy despite the distribution appearing “spread out” over [a,b].

### 4.4. Relationship to Maximum Entropy Principle

The Maximum Entropy (MaxEnt) principle and our invariant entropy framework address different questions. MaxEnt is a distribution selection principle: given constraints (e.g., fixed mean, fixed variance), it selects the distribution that maximizes entropy, yielding the “least biased” distribution compatible with the constraints. Invariant entropy is an entropy measurement principle: given a distribution (known or empirically observed), it computes an entropy value that is invariant to measurement units. These frameworks are complementary rather than competing.

The MaxEnt principle yields different distributions depending on the constraint structure:

Bounded support constraint: Among all distributions with support contained in a fixed interval [a,b], the uniform distribution U(a,b) maximizes differential entropy:

$$h_{\mathrm{uniform}}=\log\left(b-a\right)$$

For the uniform distribution, the variance $\sigma^{2}={\left(b-a\right)}^{2}/12$ is determined by the interval bounds, not independently specified.

Fixed variance constraint on R: Among all distributions on R with fixed variance $\sigma^{2}$, the normal distribution N(μ,σ) maximizes differential entropy:

$$h_{\mathrm{normal}}=\frac{1}{2}\log\left(2\pi e\sigma^{2}\right)$$

This is fundamentally different: the domain is unbounded, and variance is an independent constraint.

From the invariant entropy perspective, all uniform distributions yield $h_{c}\approx 1.060$ and all normal distributions yield $h_{c}\approx 1.150$, regardless of their parameters (Table 2). The uniform has relatively low invariant entropy (1.060). This reflects what the invariant measure captures: the median nearest-neighbor distance reflects the typical local spacing of points. For a uniform distribution, this spacing is highly regular—when we normalize by m(X), all points lie within a predictable range relative to their typical spacing. In contrast, heavy-tailed distributions like Cauchy and Levy exhibit extreme variability in local density: some regions have tightly clustered points while others are sparse. This variability persists after normalization, yielding higher invariant entropy. Distributions with high standard differential entropy (given appropriate constraints) also tend to have high invariant entropy, suggesting consistency between the two frameworks rather than competition.

### 4.5. Connections to Information Geometry

From the perspective of information geometry [16], our approach can be understood as choosing a coordinate system on the manifold of probability distributions. The Fisher information metric provides a natural Riemannian structure on this manifold, and geodesics correspond to exponential families.

The invariant measure m(X) defines a natural coordinate chart that makes entropy calculations coordinate-independent, analogous to working in canonical coordinates in differential geometry. For a family of distributions $\left\{p_{\theta }:\theta \in \Theta \right\}$, the Fisher metric is:

(33) $$g_{ij}\left(\theta \right)=E\left[\frac{\partial \log p_{\theta }}{\partial \theta_{i}}\frac{\partial \log p_{\theta }}{\partial \theta_{j}}\right]$$

For location-scale families $p_{\mu ,\sigma }\left(x\right)=\frac{1}{\sigma }p_{0}\left(\frac{x-\mu }{\sigma }\right)$, the invariant measure removes the (μ,σ) dependence, effectively projecting onto the “shape manifold” of distributions. Future work could explore whether there is a canonical connection between our invariant measure and the Fisher–Rao metric, potentially leading to a geometric interpretation of the LDDP as arc length on the shape manifold.

### 4.6. Limitations and Future Directions

1. Beyond affine transformations: Our current framework handles affine transformations (x,y)→(ax+b,cy+d). Extending to general diffeomorphisms $\phi :R^{d}\to R^{d}$ would require incorporating the Jacobian determinant |detDϕ|. The invariant measure would need to transform as $m\left(\phi \left(X\right)\right)=m\left(X\right)\cdot {\left|\det D\phi \right|}^{1/d}$ to preserve invariance. This generalization could connect to the theory of differential forms and volume elements in differential geometry.

2. Theoretical optimality of the median: While our empirical results demonstrate that the median of nearest-neighbor distances avoids negative entropy and satisfies all required invariance properties, a complete mathematical characterization remains to be established. The median is optimal as an $L^{1}$ center (minimizing $\sum |d_{i}-c|$), while the mean is optimal as an $L^{2}$ center (minimizing $\sum {\left(d_{i}-c\right)}^{2}$). Different $L^{p}$ centers induce different geometries on the distance distribution, each preserving the geometric invariance properties but potentially yielding different entropy values. Establishing the minimax optimality of the median among all $L^{p}$ centers for minimizing the probability of negative entropy across relevant distribution classes, and characterizing the tail conditions under which $P(h_{c}<0)\to 0$ as N→∞, would provide a rigorous theoretical foundation. Such an analysis could parallel the development of M-estimators in robust statistics, potentially establishing conditions under which the $L^{1}$ geometry provides the natural reference scale for entropy measurement.

3. Connection to rate-distortion theory: Rate-distortion theory [12] involves minimizing I(X;Y) subject to distortion constraints E[d(X,Y)]≤D. The invariant MI might provide new insights into scale-invariant coding schemes where the distortion metric itself adapts to the data scale. This could have applications in lossy compression where preservation of “shape” is more important than absolute accuracy.

4. Applications in causality: Invariance under interventions is central to causal inference. Pearl’s do-calculus and the invariance principle of Peters et al. both rely on identifying relationships that remain stable across environments. Our scale-invariant MI might help identify causal relationships that persist across different measurement scales or units, potentially improving causal discovery algorithms when variables are measured inconsistently across datasets.

5. Extensions to discrete–continuous mixtures: Many real-world datasets contain both discrete and continuous variables. The MI between discrete and continuous variables is well-defined, but estimating it is challenging [2]. The invariant measure framework could potentially be extended to mixed data types by defining appropriate reference measures for discrete components.

## 5. Conclusions

This work establishes a rigorous theoretical foundation for invariant entropy estimation by connecting Jaynes’s limiting density of discrete points to the Kullback–Leibler divergence framework. By defining the invariant measure m(X) as the median of nearest-neighbor distances and proving that $h_{c}\left(X\right)=h\left(X/m\left(X\right)\right)$ is truly scale-invariant, we provide the first practical method for computing Jaynes’s LDDP. The approach extends naturally to multivariate settings and demonstrates superior performance compared to standard methods, particularly avoiding catastrophic failures when variables have disparate scales.

The invariant entropy represents a logical evolution of Shannon’s information theory for continuous variables. Just as Shannon entropy for discrete variables is invariant to relabeling of outcomes, our invariant entropy for continuous variables is invariant to rescaling of measurement units. This makes it a more natural measure of uncertainty for physical quantities that can be measured in different units—temperature in Celsius versus Fahrenheit, distance in meters versus feet, concentration in molarity versus parts-per-million.

Beyond practical utility, the invariant entropy offers theoretical insights. Table 2 reveals that the arcsine distribution, despite appearing spread over an interval, has the lowest invariant entropy due to boundary concentration effects visible at the nearest-neighbor scale. Conversely, heavy-tailed distributions like Cauchy and Levy have high invariant entropy, reflecting fundamental unpredictability even at local scales. These observations suggest that invariant entropy captures the intrinsic properties of distribution families independent of parametrization.

By grounding this concept in the well-established KL divergence framework, we provide both theoretical justification and practical tools for scale-invariant information-theoretic analysis. The connection to information geometry suggests deeper links between invariant measures and the Fisher–Rao metric on distribution manifolds, opening avenues for future research.

We hope this work will enable new applications across diverse scientific fields where scale invariance is essential: feature selection in machine learning with mixed-unit data, network inference in systems biology where genes have vastly different expression scales, time series analysis comparing signals with different amplitudes, and causal discovery across heterogeneous datasets. Open-source implementations in Julia (EntropyInvariant.jl) and Python (entropy_invariant) make these methods readily accessible to the research community.

## Figures and Tables

**Table 1:** Comparison of entropy values using mean versus median invariant measure for exponential and normal distributions. We use standard parameterizations: E(λ) with density $\lambda e^{-\lambda x}$ for x≥0 (mean =1/λ); N(μ,σ) with density $\frac{1}{\sigma \sqrt{2\pi }}e^{-{\left(x-\mu \right)}^{2}/\left(2\sigma^{2}\right)}$. Numerical values use λ=1 and N(0,1).

Entropy (Mean)	Entropy (Median)
$h_{c_{\mathrm{mean}}}\left(E\left(\lambda \right)\right)=-0.06$	$h_{c_{\mathrm{median}}}\left(E\left(\lambda \right)\right)=1.22$
$h_{c_{\mathrm{mean}}}\left(N\left(\mu ,\sigma \right)\right)=-0.80$	$h_{c_{\mathrm{median}}}\left(N\left(\mu ,\sigma \right)\right)=1.14$

**Table 2:** Invariant entropy for common distributions. Each value represents the mean ± standard deviation over 100 simulations with 10,000 samples each. For bounded distributions: Arcsine(0,1), Uniform(0,1), Semicircle with r=1, Triangular(0,1,0.5), Cosine(μ=0.5,s=1). For unbounded distributions: standard parameters were used (e.g., N(0,1), E(1)). Due to the invariance property, these values remain constant for any choice of location and scale parameters within each distribution family.

Distribution	Invariant Entropy
Arcsine (a,b)	1.008 ± 0.006
Uniform (a,b)	1.060 ± 0.005
Semicircle (r)	1.073 ± 0.005
Triangular (μ,σ)	1.106 ± 0.005
Cosine (μ,σ)	1.114 ± 0.005
Normal (μ,σ)	1.150 ± 0.005
Rayleigh (σ)	1.135 ± 0.005
Chi (ν)	1.149 ± 0.005
Gumbel (μ,σ)	1.174 ± 0.005
Logistic (ν)	1.184 ± 0.005
Exponential (θ)	1.227 ± 0.005
Laplace (μ,σ)	1.227 ± 0.005
Cauchy (μ,σ)	1.517 ± 0.006
Levy (μ,σ)	1.973 ± 0.008

## Data Availability

The methods described in this paper are implemented in two open-source packages: the EntropyInvariant.jl Julia package (https://github.com/Entropy-Invariant/EntropyInvariant.jl (accessed on 28 January 2026)) and the entropy_invariant Python package (available via PyPI: pip install entropy_invariant). Both implementations provide identical functionality and produce numerically equivalent results. All simulation code for reproducing the figures is provided in the Appendix A.

## Abbreviations

    The following abbreviations are used in this manuscript:

LDDP	Limiting Density of Discrete Points
kNN	k-Nearest Neighbor
MI	Mutual Information
KL	Kullback–Leibler
KDE	Kernel Density Estimators
IQR	Inter-Quartile Range

## Appendix A. Computational Details and Package Usage

The calculations and examples in this manuscript have been implemented using the Julia programming language and the EntropyInvariant.jl package (https://github.com/Entropy-Invariant/EntropyInvariant.jl (accessed on 28 January 2026)). The package is registered through the Julia package manager and can be installed as follows:

julia> ] add EntropyInvariant

### Appendix A.1. Main Entropy Function

The EntropyInvariant package provides a unified interface for computing entropy using histogram, kNN, and invariant methods:

entropy(mat::Matrix{<:Real};
      method::String = "inv",
      nbins::Int = 10,
      k::Int = 3,
      base::Real = e,
      verbose::Bool = false,
      degenerate::Bool = false,
      dim::Int = 1
      )::Real

The method parameter selects the estimation method: “inv” for invariant estimation (default), “knn” for standard kNN method, or “histogram” for histogram-based estimation.

### Appendix A.2. Algorithmic Details of Invariant Measure Estimation

The computation of the invariant entropy $h_{c}\left(X\right)=h\left(X/m\left(X\right)\right)$ requires estimating the invariant measure m(X) from data. For a sample $\left\{x_{1},x_{2},\ldots ,x_{n}\right\}$, the algorithm proceeds as follows:

Step 1: Compute nearest-neighbor distances. For each dimension *d*, sort the data and compute the distance from each point to its nearest neighbor:

(A1) $$d_{i}=\min_{j\neq i}\left|x_{i}^{\left(d\right)}-x_{j}^{\left(d\right)}\right|,\;i=1,\ldots ,n$$

Step 2: Extract the invariant measure. The median of the distance distribution provides a robust estimate of the characteristic scale:

(A2) $$m\left(X^{\left(d\right)}\right)=\mathrm{median}\left(\left\{d_{1},d_{2},\ldots ,d_{n}\right\}\right)$$

The median is preferred over the mean because it satisfies the required invariance properties and avoids negative entropy values. For the exponential distribution, the mean-based measure yields $h_{c}=-0.06$ (negative), whereas the median-based measure gives $h_{c}=1.22$ (positive).

Step 3: Normalize the data. Each dimension is divided by its invariant measure:

(A3) $$X^{\prime (d)}=\frac{X^{\left(d\right)}}{m(X^{\left(d\right)})}$$

Step 4: Apply kNN entropy estimator. The normalized data $X^{\prime }$ is used in the standard Kraskov estimator, yielding $h_{c}\left(X\right)=h_{\mathrm{kNN}}\left(X^{\prime }\right)$.

This procedure ensures that $h_{c}\left(aX+b\right)=h_{c}\left(X\right)$ for any affine transformation, as proven in Theorem 1.

### Appendix A.3. Python Implementation

A Python port providing identical functionality is available via PyPI:

pip install entropy_invariant

The API mirrors the Julia interface and produces numerically identical results. Example usage:

from entropy_invariant import entropy, mutual_information
h = entropy(data, method="inv", k=3)

### Appendix A.4. Additional Functions

The package provides additional information-theoretic quantities beyond those demonstrated in this appendix: conditional_entropy, conditional_mutual_information, normalized_mutual_information, interaction_information, and Partial Information Decomposition functions (redundancy, unique, synergy). Complete documentation is available in the package repositories.

Basic usage examples:

julia> using EntropyInvariant
  julia> data = rand(100, 2)  # 100 points in 2D

# Using the invariant method (default)
  julia> h_inv = entropy(data, method="inv", k=3)

# Using the kNN method
  julia> h_knn = entropy(data, method="knn", k=5)

# Using the histogram method
  julia> h_hist = entropy(data, method="histogram", nbins=10)

### Appendix A.5. Scale Invariance Demonstration

The following example demonstrates the scale invariance property of the invariant entropy:

julia> using EntropyInvariant, Random
  julia> Random.seed!(42)
  julia> n = 1000
  julia> p1 = rand(n)

julia> println("Entropy invariant:")
  julia> println(entropy(p1))
  julia> println(entropy(1e5 * p1 .- 123.465))
  julia> println(entropy(1e-5 * p1 .+ 654.321))

Entropy invariant:
  1.087225165954103
  1.087225165954023
  1.087224434765357

All three entropy values are essentially identical despite dramatic scale transformations ($\times 10^{5}$ and $\times 10^{-5}$) and translations, confirming the invariance property stated in Theorem 1.

### Appendix A.6. Reproducing Figure 1: Invariant Entropy Estimation

The following code reproduces the results shown in Figure 1:

julia> using EntropyInvariant, Random, Distributions

julia> Random.seed!(42)
  julia> N = 100:500:10,100  # sample sizes
  julia> KNN = 3:3:30  # k values
  julia> NB = 100  # number of simulations

# Initialize storage arrays
  julia> inv_knn_nor = zeros(NB, length(KNN), length(N))
  julia> inv_knn_uni = zeros(NB, length(KNN), length(N))
  julia> inv_knn_exp = zeros(NB, length(KNN), length(N))

# Run simulations
  julia> for nb in 1:NB
         for (idx_knn, k) in enumerate(KNN)
            for (idx_n, n) in enumerate(N)
               # Normal distribution
               nor_ = rand(Normal(0, 1), n)
               inv_knn_nor[nb, idx_knn, idx_n] =
                   entropy(nor_, k=k, method="inv")

             # Uniform distribution
               uni_ = rand(Uniform(0, 1), n)
               inv_knn_uni[nb, idx_knn, idx_n] =
                   entropy(uni_, k=k, method="inv")

             # Exponential distribution
               exp_ = rand(Exponential(1), n)
               inv_knn_exp[nb, idx_knn, idx_n] =
                   entropy(exp_, k=k, method="inv")
            end
         end
      end

# Compute means and standard deviations
  julia> mean_nor = mean(inv_knn_nor, dims=1)
  julia> std_nor = std(inv_knn_nor, dims=1)
  # Similar for uniform and exponential...

### Appendix A.7. Mutual Information Estimation

The package provides a mutual information function that uses the invariant entropy by default:

mutual_information(x::Vector{<:Real},
            y::Vector{<:Real};
            method::String = "inv",
            k::Int = 3,
            nbins::Int = 10)::Real

Example: Computing MI between independent variables with extreme scale differences

julia> using EntropyInvariant, Random, Distributions

julia> Random.seed!(42)
  julia> n = 10,000

# Three independent variables with different scales
  julia> X = rand(Normal(0, 0.01), n)
  julia> Y = rand(Normal(0, 1), n)
  julia> Z = rand(Normal(0, 100), n)

# Compute mutual information (should be \approx 0)
  julia> MI_XY_inv = mutual_information(X, Y, method="inv", k=5)
  julia> MI_XZ_inv = mutual_information(X, Z, method="inv", k=5)
  julia> MI_YZ_inv = mutual_information(Y, Z, method="inv", k=5)

julia> println("MI(X;Y) = ", MI_XY_inv)
  julia> println("MI(X;Z) = ", MI_XZ_inv)
  julia> println("MI(Y;Z) = ", MI_YZ_inv)

MI(X;Y) = 0.0132
  MI(X;Z) = 0.0116
  MI(Y;Z) = 0.0115

Compare with standard kNN method showing catastrophic failure:

julia> MI_XY_knn = mutual_information(X, Y, method="knn", k=5)
  julia> MI_XZ_knn = mutual_information(X, Z, method="knn", k=5)
  julia> MI_YZ_knn = mutual_information(Y, Z, method="knn", k=5)

julia> println("MI(X;Y) = ", MI_XY_knn)
  julia> println("MI(X;Z) = ", MI_XZ_knn)
  julia> println("MI(Y;Z) = ", MI_YZ_knn)

MI(X;Y) = 0.0277
  MI(X;Z) = -1.3216 # Catastrophic failure: negative MI!
  MI(Y;Z) = 0.0226

The negative MI value for I(X;Z) in the standard kNN method demonstrates how severely scale differences can corrupt estimation. This physically impossible result (MI must be non-negative) occurs because the kNN estimator computes joint nearest-neighbor distances in the (X,Z) space, where the 100-fold scale difference causes *Z* to dominate the distance metric. The invariant method avoids this by normalizing each variable to its intrinsic scale before computing joint entropy.

### Appendix A.8. Reproducing Figure 2: MI Comparison

The following code generates the data for Figure 2, comparing histogram, kNN, and invariant methods:

julia> using EntropyInvariant, Random, Distributions

julia> Random.seed!(42)
  julia> N = 100:500:10,100
  julia> BINS = 5:5:50
  julia> KNN = 3:3:30
  julia> NB = 100

# Initialize storage for histogram method
  julia> mi_hist_XY = zeros(NB, length(BINS), length(N))
  julia> mi_hist_XZ = zeros(NB, length(BINS), length(N))
  julia> mi_hist_YZ = zeros(NB, length(BINS), length(N))

julia> for nb in 1:NB
         for (idx_bins, bins) in enumerate(BINS)
            for (idx_n, n) in enumerate(N)
               X = rand(Normal(0, 0.1), n)
               Y = rand(Normal(0, 1), n)
               Z = rand(Normal(0, 10), n)

             mi_hist_XY[nb, idx_bins, idx_n] =
                 mutual_information(X, Y,
                   method="histogram", nbins=bins)
               mi_hist_XZ[nb, idx_bins, idx_n] =
                 mutual_information(X, Z,
                   method="histogram", nbins=bins)
               mi_hist_YZ[nb, idx_bins, idx_n] =
                 mutual_information(Y, Z,
                   method="histogram", nbins=bins)
            end
         end
      end

# Similar loops for kNN and invariant methods...
